# Participation and Experiences in Extracurricular Activities for Autistic and Neurotypical Children

**DOI:** 10.1007/s10803-023-06142-z

**Published:** 2023-10-13

**Authors:** Callyn Farrell, Virginia Slaughter, Tomomi McAuliffe, Aisling Mulvihill

**Affiliations:** 1 School of Psychology, The University of Queensland, Brisbane, QLD Australia; 2 School of Health and Rehabilitation Sciences, The University of Queensland, Brisbane, QLD Australia; 3 School of Psychology, The University of Queensland, St. Lucia Campus, Level Three, McElwain Building, Brisbane, QLD 4072 Australia

**Keywords:** Autism, Leisure activities, Extracurricular activities, Participation, Barriers, Facilitators

## Abstract

Participation in Organised Extracurricular Social Activities (OESA) can provide positive outcomes for children. This study investigated whether children aged 4 to 12 years diagnosed with autism differ in their OESA participation and experience compared to neurotypical peers. Parents of autistic children (*n* = 35) and those of neurotypical peers (*n* = 171) responded to questions that asked them to reflect on their child’s participation and experiences in OESAs. Parents of autistic children reported significantly less OESA participation compared to parents of neurotypical children. Additionally, when evaluating factors that facilitated OESA participation, parents of autistic children rated their child’s individual abilities and behaviour, the OESA’s features, and the social environment less positively, compared to parents of neurotypical children. OESA participation and experiences differ for autistic and neurotypical children. This study identifies factors that can be adjusted to mitigate this difference.

## Introduction

Children can develop autonomy, a sense of belonging, and self-efficacy through participation in leisure activities, in turn, improving their subjective well-being (Hocking, 2009). Organised Extracurricular Social Activities (OESAs) are leisure activities for children that are organised, extracurricular, and social in nature, capturing a broad range of activities (e.g., sports, dance classes, art classes). Participation in OESAs is associated with a range of positive developmental and psychosocial outcomes for children, and these benefits accrue both to neurotypical children (McCabe et al. 2016; Peck et al. 2008, May et al. 2021, Bohnert et al. 2019).

Autism is a neurodevelopmental diagnosis, typically diagnosed in childhood. Differences in social interaction and relationships, nonverbal and verbal communication, atypical expressions in sensory processing, and a restricted range of interests and activities characterise autism (American Psychiatric Association, 2013 ). Collectively these experiences can have variable and significant impacts on a child’s daily functioning, engagement, and participation (Mattinson et al. 2018 ).

Global autism prevalence seems to be rising, with an estimated one child in every 36 diagnosed in the US (Centers for Disease Control and Prevention, 2023). In Australia, the most recent nationwide data indicate approximately one in 160 children between the ages of 6 and 12 years is diagnosed with autism (Wigston et al., 2017). Assistance with self-care, daily life tasks, mobility, communication, and emotion regulation is required by approximately one-third of individuals living with autism (Australian Bureau of Statistics [ABS], 2012). Additionally, autistic children and their families can be viewed negatively, and experience judgement and misunderstanding within society, leading to experiences of stigmatisation (Aube et al., 2021; Broady et al., 2017; Mauzumder & Thompson-Hodgetts, 2019). Therefore, both personal and societal characteristics associated with autism may negatively impact participation and experience in OESAs. It is important that researchers and practitioners alike understand these impacts to foster healthy and positive participation and experiences in leisure activities, such as OESAs.

The term OESA inclusively broadens the scope of leisure activities for children that have been investigated in previous research and spotlights activities that are non-solitary in nature. Previously, OESAs have been represented by various terms such as physical activities, extracurricular activities, leisure activities, organised activities, out of school recreation/leisure, and after school activities. Here we offer a precise definition: OESAs are formally ***organised*** and structured activities for children where an adult, coach or program leader provides supervision. OESAs are voluntarily attended and take place regularly for an extended period, such as a season. OESAs are ***extracurricular*** as they are independent of formal schooling and typically organised outside of school hours. Importantly, OESAs refer to activities that are non-solitary, therefore providing ***social*** opportunities to interact, connect, and learn with peers, friends, or other individuals. OESAs capture a wide range of ***activities***, for example, sporting, social, physical, academic, artistic, religious, and community-based activities.

Research has established positive relationships between increased participation in OESAs and positive psychological, behavioural, social and academic outcomes (Hynes & Block, 2022; McCabe et al. 2016; Peck et al. 2008; Schaefer et al. 2011). For example, Fredricks and Eccles (2005) found that those adolescents who participated in OESAs self-reported higher academic performance and greater psychological well-being. Similarly, OESA participation has been found to have positive peer outcomes for school-aged children, including increased connectedness, well-being, social and emotional adjustment, and decreased loneliness (Oberle et al. 2019). Therefore, participation in OESAs appears to present a broad range of transferrable benefits for daily life.

Similar to their neurotypical peers, children with developmental diagnoses can derive wide-ranging benefits from OESA participation. Research indicates that physical activity participation for children with developmental diagnoses may provide health-related benefits, for example, increased cardiovascular fitness and gross motor functioning (García-Hermoso et al. 2021; Huang et al. 2020). Further, participation in OESAs, such as dance has been found to offer a broad range of benefits, including cognitive, psychological and social outcomes, for children and adolescents with disabilities (May et al. 2021). For autistic children, continuous participation in physical activities has also been shown to reduce restricted, stereotyped, and repetitive behaviours (Rosenthal-Malek & Mitchell, 1997). Bohnert et al. (2019) found that greater breadth of organised activity participation was associated with improved socio-emotional adjustment for autistic adolescents.

Notwithstanding the benefits of OESA participation, children with disabilities generally experience reduced participation in OESAs (Law et al. 2011; King et al. 2010; Engel-Yeger et al. 2009 ) and autistic children are no exception to these findings. Autistic children may participate in fewer types of OESAs for a lesser time (Bandini et al. 2013). This may limit their opportunities to derive the wide-ranging psychosocial benefits offered by OESA participation and experience. Several studies have investigated the barriers to OESA participation for autistic children. Much of this research highlights child factors related to an autism diagnosis, including communication and social skill deficits (Müller et al. 2008 ), preferences for sedentary pursuits (i.e., ‘screen time’; Arkesteyn et al. 2023; Obrusnikova & Cavalier, 2011), narrowly focused interests (Obrusnikova & Dillon, 2011 ), and fine and gross motor skill deficits (Fournier et al. 2010).

In an investigation of barriers to OESA participation for autistic children, Must et al. (2015) surveyed parents of 58 neurotypical children and 53 autistic children aged 3 to 11years using questions that assessed perceived barriers to their child’s participation in physical activities. The following categories were assessed; child/family barriers, social barriers and community barriers. Parents reported that each of these barrier categories limited physical activity participation for autistic children compared to neurotypical children. Thus, autistic children may experience a range of barriers to OESA participation that may hinder access to the vital psychosocial developmental benefits offered.

Existing research on OESA participation for autistic children is limited in several ways. Most published data represents a United States context and prioritises extracurricular physical activities (Obrusnikova & Cavalier, 2011; Must et al. 2015; Obrusnikova & Miccinello, 2012), with few studies exploring autistic children’s experiences in a broader range of OESAs (see Bohnert et al. 2019). This approach fails to consider that autistic children may participate in and experience non-physical activities differently from how they participate and experience physical activities. Another limitation is that previous measures asked parents to report on their child’s OESA participation generally (Must et al. 2015) or within a specific timeframe (Bohnert et al. 2019) but neglect to investigate parental perspectives of their child’s participation specific to a child’s level of participation. It is conceivable that parental perspectives regarding barriers and facilitators may differ according to whether their autistic child withdrew from or sustained participation in an OESA. Most importantly, existing research focuses primarily on barriers to OESA participation, with little consideration of potential facilitators (Shields et al., 2012).

Research into the experiences of individuals with a disability is often informed by either a medical or social model of disability. A medical model considers disability to be a medical or individual phenomenon that subsequently leads to impairments in body structures or functions whereas the social model considers disability as a construct enforced upon society’s differential impairments (Haegle & Hodge, 2016). In isolation, medical or social models of disability can provide relevant insights. However, they offer narrow views of individualised and complex experiences.

In contrast, a biopsychosocial model moves beyond a dichotomous view of disability and accounts for the relevant components of both the social and medical models by conceptualising an individual’s disability and social context as one (Castro et al. 2011). Grounded within the biopsychosocial model of disability, the ICF-CY (World Health Organisation, 2007) provides a multilayered understanding of an individual’s functioning, specifically focusing on participation in daily activities (Adolfsson et al. 2011 ). Therefore, this offers a comprehensive framework to investigate OESA participation for autistic children.

**Fig. 1:**
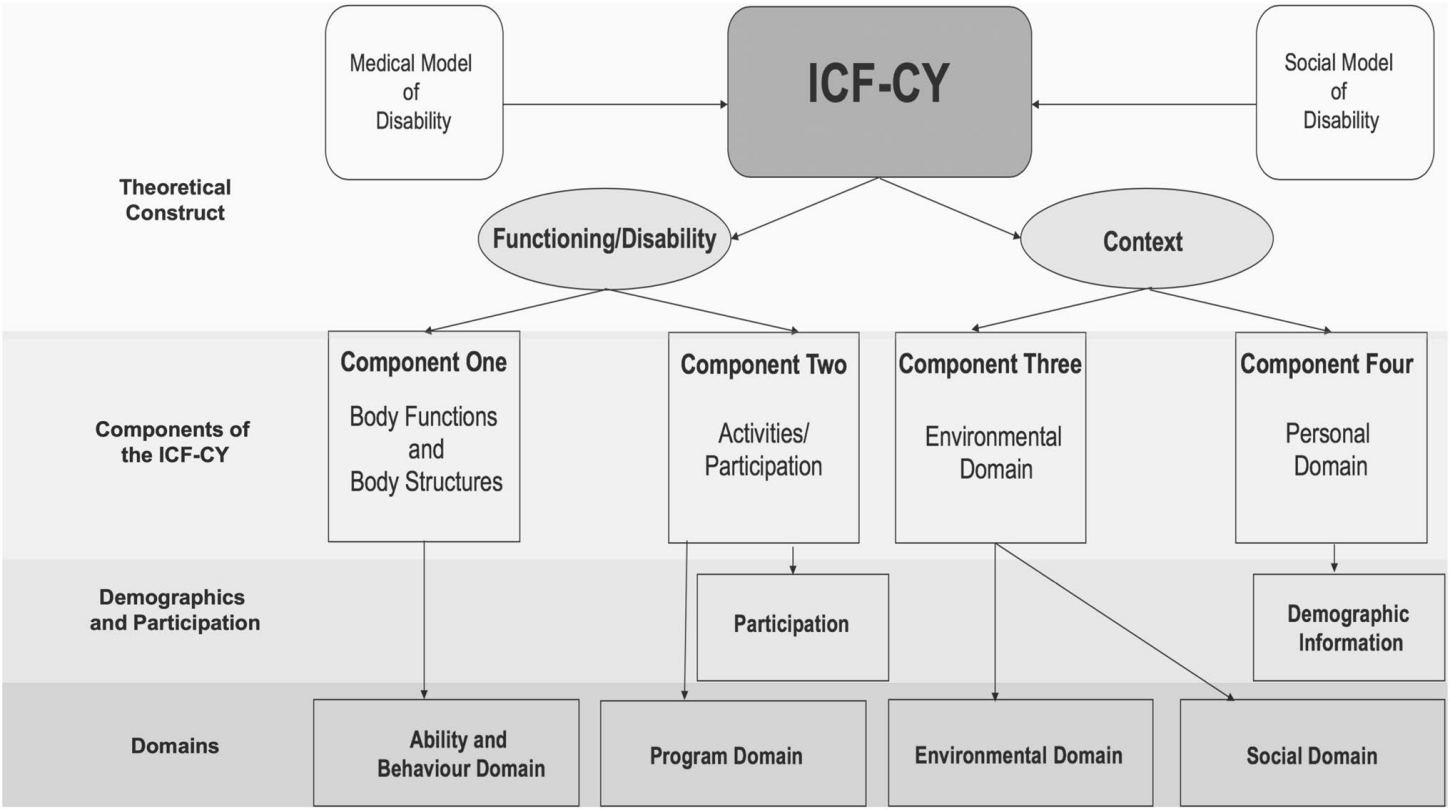
A structural representation of the ICF-CYs theoretical construct, its components, and how they inform the present study

Two over-arching categories create the ICF-CY; Functioning/Disability and Context (Adolfsson et al. 2011 ). These are further subdivided into four components (see Fig. 1). Component One, The Body, considers a child’s psychological, physical, and sensory functioning and body structures (Ibragimova et al. 2009). Component Two, Activities and Participation, encompasses all daily activities. Component Three, Environmental Factors, includes social, attitudinal, and physical aspects of a child’s environment. Component Four, Personal Factors, is captured by demographic information (Adolfsson et al. 2011 ). Therefore, the ICF-CY considers how all aspects of a child’s functioning, disability, and context, impact their participation and experience in activities of daily living.

This study aimed to understand a child’s participation in OESAs across all four ICF-CY components. Hence, the ICF-CY informed the questions presented to parents. These questions considered participation across three levels of engagement; fully engaged, partially engaged, and considered engagement - that is OESAs that were contemplated but for which there was no actual participation. Additionally, experiences were assessed considering barriers and facilitators across four domains (ability and behaviour, social, program, and environmental).

To our knowledge, this is the first study to comprehensively investigate OESA participation differences between autistic and neurotypical children in an Australian context. We aimed to examine several exploratory research questions. Firstly, which OESA categories (as per those used by Bohnert and colleagues (2019): Religious, Academic, the Arts, Sports, and Community/Service) are most reported on at each level of participation and does this differ between autistic and neurotypical children? Secondly, how does OESA participation intensity, breadth, and number of OESAs compare between autistic and neurotypical children? Lastly, what are the experienced barriers and facilitators at each of the three levels of participation, and does this differ between autistic and neurotypical children?

## Method

### Participants

Participants were 247 Australian parents of children aged 4- to 12-years, recruited through The Early Cognitive Development Centre. This age sampling is most appropriate because school-aged children’s participation in OESAs intuitively requires parental support and involvement. Further, investigating children entering adolescence and adulthood may require different approaches (i.e., interviewing the adolescent themselves). Of the 211 responses five were removed due to an unrealistic response (e.g., reporting child age as 40-years). In Australia, children can begin formal schooling at the age of 4-years if born in or before July. Hence, parents of 4-year-old children were included in this study. Thus, the final sample consisted of 206 parent participants between 26- and 54-years (*M* = 40.53, *SD* = 5.53); parents of 35 autistic children between the ages of 4.75- and 12.50-years (*M* = 9.06, *SD* = 2.33) and 171 neurotypical children between the ages of 4.08- and 12.90-years (*M* = 8.15, *SD* = 2.63). The final sample primarily represented White, high-income earning, and educated households (see Table 1 and Appendix A for further details).

**Table 1:** Sociodemographic Characteristics of the Final Sample of Participants

Characteristic	Full Sample	
	*n*	%
Sex		
Female	190	92.23
Male	16	7.77
Caregiver Relationship		
Biological Mother	189	91.75
Biological Father	16	7.77
Adoptive/Foster Caregiver	1	0.49
Annual Household Income ($AUD after taxation)		
Under 30,000	4	1.94
30,000–50,000	4	1.94
51,000–75,000	12	5.82
76,000–99,000	21	10.19
100,000 or over	138	67.0
Preferred not to respond	27	13.11
Level of Education		
Year 10 or equivalent	2	0.97
Year 12 or equivalent	7	3.40
Certificate/Diploma	37	17.96
University Degree	70	33.98
Higher University Degree	89	43.20
Preferred not to respond	1	0.49

* N* = 206

### Procedure

Participants were recruited from the database of The Early Cognitive Development Centre. Participants were invited to participate via email and the study was hosted using Qualtrics. Parents provided informed consent before completing the study. Parents were first asked to provide demographic information regarding themselves and their child. Details regarding an autism diagnosis date and the diagnosing professional were required for those participants answering about a child diagnosed with autism. Following this, an OESA definition was presented to participants. Next, questions regarding levels of participation were presented in a fixed order. The study took participants an average of 39.62 min to complete. Participants could discontinue their participation freely and a written study debrief was provided.

### Materials

The present study used a self-report measure comprised of 147 items completed by a parent/caregiver. We collected comprehensive demographic information, information on the breadth and intensity of OESA participation and experiences across three levels.

The four components of the ICF-CY informed the content of the items within our self-report measure: (1) The Body, (2) Activities and Participation, (3) Environmental Factors, and (4) Personal Factors. The ICF-CY components map onto the content domains of the questions presented to participants (see Fig. 1). The Body component of the ICF-CY informs items within the ability and behaviour domain, which aim to capture the influence of a child’s own physical, communicative, cognitive, and behavioural abilities on their participation and experience. The Activities and Participation component of the ICF-CY informs the three levels of participation. Also, items in the program domain aim to capture the influence the OESA has on a child’s participation and experience, for example, the activities’ flexibility, behavioural accommodation, and the level of parental support for the child’s participation in the OESA. The Environmental Factors component of the ICF-CY informs the environmental domain, which aims to capture the influence of the environment the OESA takes place in, such as the physical infrastructure and the accessibility of the OESA. Also, the social domain, which aims to capture the influence social factors such as the attitudes and beliefs of others have upon a child’s participation and experience, as well as the social roles individuals play in the OESAs social environment. Personal Factors are represented by demographic information.

To gain a comprehensive understanding of OESA participation and experience, we examined facilitators and barriers to participation across three levels. Level One (fully engaged) asks parents to comment on OESAs that their child participated in regularly during the past year. Although children are likely to persist with a given OESA for several seasons/years, this level was defined over one year to support reliable recall. Specifically, at Level One we highlight the importance of capturing recent experiences to support reliable recall while still allowing parents to provide meaningful insights into their child’s OESA participation and experience. Level Two (partially engaged) asks parents to comment on OESAs that their child participated in but withdrew from over the past five years. Level Three (considered engagement) asks parents to comment on OESAs that were contemplated but for which there was no actual participation over the past five years. The last two levels of participation collect information over a five-year timeframe as parents may not have withdrawn from or considered participation in a given OESA within a single year. Therefore, a five-year recall period may increase access to information about disengagement and barriers to participation.

Parents could list no OESA if the level of participation did not apply, or a maximum of five OESAs if the level of participation was applicable. Levels One and Two of participation (fully engaged and partially engaged, respectively) comprised of three open-ended items. The first asked for the name of the OESA the child participated in. The second requested the average hours per week the activity was engaged in, and the third asked for the duration of participation in weeks or months (at Level One). While at Level Two, the third question asked for the duration of participation before termination. Lastly, Level Three (considered engagement) comprised of one open-ended item that asked the participant to list the name of an OESA they had considered for their child but not engaged in.

For each level of OESA participation, if more than one OESA was listed, participants selected one as a reference for the domain related items that followed. Next, 29 fixed-order items asked parents to rate “to what extent do you feel each of the following factors influenced participation in [the nominated OESA] …”. Therefore, the items aimed to gather information relating to a child’s experiences within a given OESA, and if these experiences acted as a barrier or facilitator. The ability and behaviour domain consisted of eight items (e.g., *my child’s behaviour*), the program domain included seven items (e.g., *opportunities to start at beginner or introductory levels*), the environmental domain included six items (e.g., *the availability of transport)*, and the social domain included eight items (e.g., *attitudes of other parents towards my child*). These items were answered on a slider bar, which provided a continuous measure of experience as negative, neutral or positive. Scores are derived from the sliding scale and range from −1.00 (*a negative influence/barrier*) to 1.00 (*a positive influence/facilitator*), with 0.00 representing a neutral influence. Thus, when a participant positioned the slider at any point of the bar, a continuous output score ranging from −1.00 to 1.00 was calculated.

#### OESA Categorisation

As per Bohnert et al. (2019), each reported OESA was assigned to one of five mutually exclusive categories: Religious, Academic, the Arts, Sports, and Community/Service. The primary nature of the activity informed the assignment of a category of the OESAs content. Authors CF and VS independently categorised all OESAs provided by participants. The resulting interrater reliability for OESA category coding was 99.00%, and the one disagreement was resolved by discussion.

#### Measurement of OESA Participation

The present study captured the breadth, the total number, and intensity of OESA participation, as well as the degree to which each domain presented as a barrier or facilitator to participation.

### OESA Breadth Score

Each participant received an OESA breadth score per level of participation. This was calculated by recording the number of different OESA categories that an individual participated in at each level.

### OESA Total Score

Parents could report between zero and five OESAs at each level of participation. An OESA total score was computed at each level by adding how many activities were listed per level.

### OESA Intensity Score

At Level One of participation (fully engaged), an OESA intensity score was calculated for each participant by multiplying the total number of hours per week by the total weeks of participation for each OESA listed. This captured the intensity of OESA participation within a one-year timeframe.

### Composite Domain Score

At each level of participation, participants rated 29 items representing four domains (i.e., ability and behaviour, program, environmental, and social) regarding a participant selected OESA. Each item was scored on a sliding scale ranging from −1.00 (*a negative influence/barrier*) to 1.00 (*a positive influence/facilitator*). Thus, a score of 0.00 represents that the item was a neutral influence on participation for participants. Item scores were summed to create four domain composite scores at each level of participation.

### Preliminary Analyses

The autistic and neurotypical groups were considered separately for preliminary analysis based on evidence that participation in physical activities could differ between groups (Bandini et al. 2013). A sex difference in sample size across groups was observed, where the autistic group comprised a greater proportion of males than females (see Appendix A). This is typical of autistic samples due to sex differences in prevalence rates, with males being 3.5 times more likely to be diagnosed with autism in Australia (ABS, 2018).

To determine if child sex or age influenced the degree that participants reported an experience domain as either a facilitator or barrier at each level of participation, a series of general linear models were computed, with between-subjects variables age (continuous, years) and sex (male, female, other) for both the autistic and neurotypical groups. Across all levels of participation, no main effects of child age or sex were found for either the autistic or the neurotypical groups, all *F* ≤ 2.32, all *p* ≥ .145, all η_*p*_^2^ ≤ 0.11. Consequently, age and sex were not considered further.

### Nature of the Data and Data Analysis

Participants were only required to report on a level of participation if it was relevant to their experience. Therefore, due to varying sample sizes across levels, analyses were conducted per level of participation.

We used nonparametric approaches for all analyses, due to differences in sample sizes between the autistic and neurotypical groups at each level of participation (see Appendix A) and because we could not assume that participants’ ratings would be normally distributed. Therefore, all descriptive statistics are reported as median scores. A series of Mann–Whitney Tests were performed to investigate whether the autistic and neurotypical groups differed on OESA measures. Subsequent Mann–Whitney Tests were performed to investigate differences in parental perceptions at the domain item level, with a significance level of *p* < .01 to control for familywise error.

## Results

### OESA Category Frequency

Groups were first compared on the OESA category (Religious, Academic, the Arts, Sports, and Community/Service) that participants chose to report on at each level of participation. A series of chi-square tests revealed no statistically significant differences between the autistic and neurotypical groups regarding the categories of OESAs reported on at any level of participation (all *p* ≥ .227; see Appendix B). Across both groups at all three levels of participation, the Sports category was the most reported.

### OESA Participation and Experiences

A series of Mann–Whitney Tests were performed to investigate whether the autistic and neurotypical groups differed on OESA breadth, the total number of reported OESAs, OESA intensity (at Level One of participation, fully engaged), and OESA experience domains across three levels of participation.

#### Level One of Participation

At Level One (fully engaged), the autistic group differed significantly from the neurotypical group regarding OESA intensity, *U* = 2051.5, *p* = .003, *r* = −.20. The autistic group completed fewer hours of OESA participation per week on average than the neurotypical group. Additionally, the autistic group (*Mdn* = 1.00, Range = 0–5) differed significantly from the neurotypical group (*Mdn* = 2.00, Range 0–5) in the total number of OESAs participated in, *U* = 1985.5, *p* = .001, *r* = −.23. Regarding the breadth of OESAs participated in, the autistic group (*Mdn* = 1.00, Range = 0–3) again differed from the neurotypical group (*Mdn* = 1.00, Range = 0–4), *U* = 2330.0, *p* = .021, *r* = −.16. These findings indicate that parents reported children with an autism diagnosis to participate in fewer OESAs, across fewer OESA categories, for fewer hours on average per week than the neurotypical group.

Regarding domain responses at level one (fully engaged), the autistic group differed significantly from the neurotypical group within the ability and behaviour domain only, *U* = 1461.5, *p* < .001, *r* = −.32. Although both groups reported this domain to be a facilitator of OESA participation, the autistic group reported it as less positive than the neurotypical group. The neurotypical and autistic groups did not differ in the program domain, *U* = 2202.5, *p* = .196, *r* = −.09, the environmental domain, *U* = 2445.5, *p* = .157, *r* = −.10, or the social domain, *U* = 2412.0, *p* = .128, *r* = −.11. These three domains were all reported as facilitators of OESA participation at Level One of participation and the scores were positive in both the neurotypical and autistic groups.

Collectively, these domain ratings indicate that from a parental perspective, the child’s ability and behaviour was a less positive facilitator of current OESA participation for autistic children compared to neurotypical children.

#### Level Two of Participation

 At Level Two (partially engaged), the autistic group (*Mdn* = 1.00, Range = 0–3) did not differ significantly from the neurotypical group (*Mdn* = 1.00, Range = 0–5) regarding the total number of OESAs participated in, *U* = 2559.5, *p* = .151, *r* = −.10. OESA breadth scores were also not significantly different between the two groups at this level (autistic: *Mdn* = 1.00, Range = 0–2; neurotypical: *Mdn* = 1.00, Range = 0–2), *U* = 2705.5, *p* = .318, *r* = −.07. These results indicate that both groups participated in and then withdrew from a similar number and range of OESAs.

Regarding factors that were perceived to influence participation, at Level Two (partially engaged), the autistic group differed significantly from the neurotypical group within the ability and behaviour domain, *U* = 708.5, *p* < .001, *r* = −.42, the program domain, *U* = 908.5, *p* = .001, *r* = −.27, and the social domain, *U* = 1134.0, *p* = .001, *r* = −.27. Importantly, these three domains were all reported as barriers to OESA participation for the autistic group, but facilitators of OESA participation for the neurotypical group. There were no significant group differences in the environmental domain, *U* = 1568.0, *p* = .183, *r* = −.11, which was perceived as a facilitator of both groups’ participation.

Thus, from a parental perspective, the child’s abilities and behaviour, social factors and program features were perceived as significant barriers to participation for the autistic group compared to the neurotypical group for OESAs that ultimately were withdrawn from.

#### Level Three of Participation

 At Level Three (considered engagement) the autistic (*Mdn* = 1.00, Range = 0–3) and neurotypical groups did not differ significantly (*Mdn* = 1.00, Range = 0–5) regarding the total number of OESA considered by parents, *U* = 2806.5, *p* = .531, *r* = −.04, nor in regards to breadth (autistic: *Mdn* = 1.00, Range = 0–2; neurotypical: *Mdn* = 1.00, Range = 0–4), *U* = 2838.5, *p* = .594, *r* = −.04. These findings indicate that both groups considered a similar number of, and a similar breadth of OESAs for their child to participate in.

Regarding domain responses at Level Three (considered engagement) the autistic group differed significantly from the neurotypical group in regard to the ability and behaviour domain, *U* = 826.0, *p* = .010, *r* = −.23, and the social domain, *U* = 705.0, *p* = .001, *r* = −.29. Parents of autistic children reported both of these domains as neutral, while parents of neurotypical children reported both domains as facilitators to OESA participation. The groups did not significantly differ in the program domain, *U* = 1030.0, *p* = .947, *r* = −.01, or the environmental domain, *U* = 1211.0, *p* = .820, *r* = −.02.

These findings indicate that parents of autistic children perceived a child’s abilities and behaviour and social factors as significantly less positive than parents of neurotypical children, when considering participation in novel OESAs.

### OESA Domain Follow-Up Analyses

Where a domain’s composite score revealed a significant difference between the autistic and neurotypical groups, a series of Mann–Whitney Tests evaluated group differences in parental perception at the domain item level. To control for familywise error in these analyses, the significance level was moved to *p* < .01.

### Level One of Participation 

The ability and behaviour domain presented a significant difference between groups. Therefore, the items that comprise this domain were investigated individually. As shown in Table 2. all items were significantly different between groups, apart from item 6. Items 1 to 5 considered communicative, motor, social and attentive abilities, item 7 children’s behaviour, and item 8 sensory preferences while participating in the OESA. For all significantly different items, the parents in the autistic group reported them as less positive for OESA participation compared to the neurotypical group.

**Table 2:** Median scores for ability and behaviour domains at levels one, two, and three of OESA participation

Item	Level One		*p*	Level Two		*p*	Level Three		*p*
	Autistic (*n *= 34)	Neurotypical (*n* = 170)		Autistic (*n *= 30)	Neurotypical (*n* = 124)		Autistic (*n *= 24)	Neurotypical (*n* = 104)	
	*Mdn* (Range)	*Mdn* (Range)		*Mdn* (Range)	*Mdn* (Range)		*Mdn* (Range)	*Mdn* (Range)	
1.My child’s communication skills	0.00 (− 1.00–1.00)	0.61 (− 0.33–1.00)	**< .001**	−0.17 (− 1.00–1.00)	0.31 (− 1.00–1.00)	**< .001**	− 0.11 (− 0.96–1.00)	0.00 (− 1.00–1.00)	**.005**
2.My child’s motor skills	0.64 (− 1.00–1.00)	0.86 (− 0.54–1.00)	**.001**	0.10 (− 1.00–1.00)	0.68 (− 0.98–1.00)	**.002**	0.43 (− 1.00–1.00)	0.38 (−1.00–1.00)	.508
3.My child’s social skills	0.00 (− 1.00–1.00)	0.71 (− 0.56–1.00)	**< .001**	−0.24 (− 1.00–1.00)	0.46 (− 1.00–1.00)	**< .001**	-0.39 (− 1.00–1.00)	0.01 (− 1.00–1.00)	**< .001**
4.My child’s coordination	0.39 (− 1.00–1.00)	0.85 (− 0.50–1.00)	**< .001**	0.11 (− 1.00–1.00)	0.60 (− 1.00–1.00)	**.001**	0.00 (− 1.00–1.00)	0.51 (− 1.00–1.00)	.015
5. My child’s attention while participating	0.00 (− 1.00–1.00)	0.76 (− 0.88–1.00)	**< .001**	0.00 (− 1.00–0.81)	0.37 (− 1.00–1.00)	**< .001**	0.00 (− 1.00–1.00)	0.00 (− 1.00–1.00)	.066
6.My child’s interests	0.76 (− 0.99–1.00)	0.81 (− 0.28–1.00)	.073	0.00 (− 1.00–1.00)	0.38 (− 1.00–1.00)	**.001**	0.49 (− 1.00–1.00)	0.44 − 1.00–1.00)	.668
7.My child’s behaviour	0.04 (− 1.00–1.00)	0.57 (− 0.68–1.00)	**< .001**	−0.09 (− 1.00–0.77)	0.02 (− 1.00–1.00)	**< .001**	0.00 (− 1.00–1.00)	0.00 (− 1.00–1.00)	**.003**
8.My child’s sensory preferences	0.00 (− 1.00–1.00)	0.35 (− 0.73–1.00)	**< .001**	−0.54 (− 1.00–0.42)	0.00 (− 1.00–1.00)	**< .001**	0.00 (− 1.00–1.00)	0.00 (− 1.00–1.00)	.021

Bolded *p* values significant at *p *< .01

### Level Two of Participation

 The ability and behaviour domain presented as significantly different between groups. As shown in Table 2., all eight items were significantly different between groups. Parents of children in the neurotypical group reported all items as facilitators to OESA participation, apart from item 8, being neutral. Items 1, 3, 7, and 8 all presented as barriers for the autistic group. Items 1 and 5 considered communicative and social abilities, item 7 children’s behaviour, and item 8 sensory preferences while participating in the OESA. Items 2, 4, 5, 6, and 7 were reported as neutral or faciliatory to OESA participation for the autistic group but all were less positive compared to the neurotypical group.

The program domain was significantly different across groups at Level Two. As shown in Table 3, subsequent analyses revealed that items 40, 43, and 44 significantly differed between the autistic and neurotypical groups, with all items being less positive for the autistic group. It is noteworthy that item 40, which examined the emotional outcomes of participating in the OESA, was seen as a barrier for the autistic group and facilitator for the neurotypical group. While item 43 considered a child’s opportunity to connect with peers within the OESA and item 44 examined the possible use flexible communication styles to suit a child’s needs or communication preferences.

**Table 3:** Domain ItemDifferences at Level Two of Participation

Program Domain	Neurotypical (*n* = 124)	Autistic (*n* = 30)	*p*
	*Mdn* (Range)	*Mdn* (Range)	
39. This activity’s accommodation of my child’s behaviour	− 0.08 (− 0.99-1.00)	0.00 (− 0.92-1.00)	0.021
40. How (name of the chosen OESA) makes my child feel	− 0.13 (− 1.00–1.00)	0.42 (− 1.00–1.00)	**< 0.001**
41. Opportunities to start (name of the chosen OESA) at beginner or introductory levels	0.53 (− 1.00–1.00)	0.56 (− 1.00–1.00)	0.118
42. The flexibility of (name of the chosen OESA)	0.00 (− 0.99-1.00)	0.00 (− 1.00–1.00)	0.093
43. The opportunity for social connection with peers, friends, parents or a buddy	0.00 (− 1.00–1.00)	0.31 (− 0.91-1.00)	**.008**
44. The use of flexible and alternative communication styles	0.00 (− 0.89-1.00)	0.00 (− 1.00–1.00)	**.007**
45. My partner, co-parent or spouse’s support of my child’s involvement in (name of the chosen OESA)	0.00^a^ (− 0.70-1.00)	0.00^b^ (− 1.00–1.00)	0.153

Bolded *p* values significant at *p* < .01

^a^*n* = 26, ^b^*n* = 117

The social domain also presented as significantly different between groups at Level Two. As shown in Table 4, items 9, 11, and 12 revealed significant differences between groups with the autistic group reporting these items as less positive than the neurotypical group, for OESAs that were withdrawn from. All three items pertained to the attitudes of other adults who may be present within the OESA context such as other parents and coach’s or program leaders.

**Table 4:** Social domain item differences at levels two and three of participation

Item	Level Two		*p*	Level Three		*p*
	Autistic (*n *= 30)	Neurotypical (*n* = 124)		Autistic (*n*= 24)	Neurotypical (*n* = 104)	
	*Mdn* (Range)	*Mdn* (Range)		*Mdn* (Range)	*Mdn* (Range)	
9.Attitudes of other parents towards me	0.00 (− 0.99–0.66)	0.00 (− 1.00–1.00)	**.007**	0.00 (− 0.96–1.00)	0.00 (− 1.00–1.00)	**< .001**
10.Attitudes of facilitators or coaches towards me	0.00 (− 1.00–1.00)	0.00 (− 1.00–1.00)	.041	0.00 (− 0.81–1.00)	0.00 (− 0.69–1.00)	**.001**
11.Attitudes of other parents towards my child	0.00 (− 1.00–0.23)	0.00 (− 1.00–1.00)	**< .001**	0.00 (− 0.83–1.00)	0.00 (− 1.00–1.00)	**< .001**
12.Attitudes of facilitators or coaches towards my child	1.00 (0.00–1.98)	1.41 (0.00–2.00)	**< .001**	0.00 (− 0.70–1.00)	0.00 (− 0.56–1.00)	**< .001**
13.The skill level of facilitators or coaches	0.00 (− 1.00–1.00)	0.43 (− 1.00–1.00)	.018	0.00 (− 0.75–1.00)	0.00 (−0.82–1.00)	**.004**
14.The individual or group nature of (name of the chosen OESA)	0.00 (− 1.00–0.95)	0.26 (− 1.00–1.00)	.094	0.00 (− 0.66–1.00)	0.00 (−1.00–1.00)	.283
15. My child’s possible exposure to bullying	0.00 (− 1.00–0.79)	0.00 (− 1.00–1.00)	.049	0.00 (− 0.68–1.00)	0.00 (-1.00-1.00)	**.004**
16.My beliefs about the value of (name of the chosen OESA) to my child	0.02 (− 0.80-0.95)	0.49 (− 1.00–1.00)	.021	0.47 (0.00–1.00)	0.38 (-0.98-1.00)	.793

Bolded *p* values significant at *p *< .01

### Level Three of Participation

 The ability and behaviour domain was significantly different between groups. As shown in Table 2., items 1, 3, and 7 were rated significantly less positively by the autistic group. Notably, item 1 (communication skills) was reported as a barrier for the autistic group but neutral for the neurotypical group. Similarly, item 3 (social skills) was reported as a barrier for the autistic group but a facilitator for the neurotypical group.

The social domain was also found to be significantly different between groups at Level Three. As shown in Table 4, items 9, 13 and 15 which included the attitudes of other parents towards the participant, the skill level of coaches, and parents’ perceived risk of bullying towards their child, were all significantly less positive for the autistic group.

## Discussion

The present study offers uniquely comprehensive evidence regarding OESA participation for autistic children compared to neurotypical children, across three levels of participation. The use of the ICF-CY framework was crucial in obtaining this evidence due to its consideration of the biopsychosocial elements of an individual’s context (Adolfsson et al. 2011). For example, we were able to examine not only the individual abilities and behavior of children but also the environmental factors that may facilitate or create barriers to participation in OESAs. This comprehensive approach allowed us to identify differences in participation levels and experiences between autistic and neurotypical children beyond differences related simply to presence or absence of an autism diagnosis.

### OESA Participation

No differences were identified between the autistic and neurotypical groups regarding the category of OESA reported on at any level of participation. Across both groups, at all levels, the Sports category was the most reported on, and this finding is consistent with Bohnert et al. (2019). This finding is encouraging as participation in sports for children and adolescents is associated with improved health outcomes and psychosocial well-being in a recent meta-analysis (see Eime et al. 2013). As the Sports category was the most reported on by both groups, future investigations may wish to explore what drives the appeal of sport OESAs for both autistic and neurotypical children, for instance the relative importance of physical, competitive and social aspects of the activities.

At Level One of participation, autistic children were reported by their parents to participate in significantly fewer OESAs, of less breadth and intensity than neurotypical children. This is concerning as greater breadth of OESA participation has been associated with improved socio-emotional adjustment for autistic children (Bohnert et al. 2019). By nature of their limited participation in a range of OESAs, autistic children may not receive the potential socio-emotional benefits of OESA participation. However, at Levels Two and Three, neither the number nor breadth of OESAs differed between groups. Therefore, parents of autistic children attempt and consider OESAs for their child at the same rate as parents of neurotypical children. Thus, the availability of or motivation to include an autistic child in OESAs may not be the most salient barrier to this population’s participation.

### Facilitators and Barriers to OESA Participation

Overall, this study’s findings indicate that social and program related domains may explain reduced OESA participation for autistic children to a greater extent than individual ability and behaviour related challenges. It is likely that this pattern of results became evident because we partitioned experience into three levels of participation.

### Limitations and Future Directions

The present study has some limitations. Despite efforts to recruit a socio-demographically diverse sample, respondents were predominantly high-income earning, highly educated, and White, across both groups. This may limit generalisability to more diverse populations. Our autistic sample size was relatively small and therefore may not represent OESA participation and experiences for all autistic children and their parents. Therefore, future studies should aim to recruit larger sample sizes of increased diversity to assess the generalisability of the findings reported here.

Furthermore, autism severity was not measured in this study. Higher autism severity scores on the Autism Diagnostic Observation Schedule (ADOS) have been shown to impact a child’s coordination and communication adversely (Beurkens et al. 2013 ), two components considered by the ability and behaviour domain. Wigston et al. (2017) found that autism severity was indeed correlated with fewer OESAs participated in. Additionally, we did not employ a measure of diagnostic characterisation in the present study. Rather, we relied on parental report of the date and the professional responsible for a child’s autism diagnosis. Therefore, the autistic sample represented here may have not included or under-represented children with profound autism, for example (Hughes et al. 2023). Future research should include measures of autism severity and diagnostic characterisation to more fully characterise OESA participation for autistic children.

Another limitation is that the data relied on parent report. Participants were required to recall activities their child had participated in as many as five years ago, possibly leading to under or over-reporting. Hence, further research should aim to explore OESA participation naturalistically where possible, as these observations may contribute essential understandings regarding OESA participation for autistic children.

Notably, for parents of neurotypical children, all domains were reported as continually facilitative of, or neutral to OESA participation and consideration. Future research should investigate OESA participation barriers at Levels Two and Three for neurotypical children. It is clear neurotypical children also withdraw from, or do not engage with OESAs at these levels. The quantitative measures used in this study may not have fully captured why this is the case. Further, as highlighted by Johnson (2009), reduced OESA participation is experienced by children with a variety of developmental disabilities. The domain questions formulated using the ICF-CY for the present study may be valuable for future research investigating OESA participation among these populations.

Finally, due to its cross-sectional design, the present study captures the perception of experiences in OESAs from a single time point. In future, longitudinal studies will be of value to the field.

### Practical Implications

As highlighted previously, the positive benefits of OESA participation include increased cardiovascular fitness and gross motor functioning (García-Hermoso et al. 2021 ; Huang et al. 2020), as well as positive cognitive, psychological, and social outcomes (May et al. 2021 ). However, for those children who experience limited participation in OESAs, these benefits may not accrue to the same extent. Specifically, based on the results reported here, it appears that autistic children may be selectively at risk of missing out on the positive benefits offered by OESA participation. While this risk may be partly attributable to a child’s individual abilities and behaviours, the current study indicates that program features of the OESA and the social environment in which an OESA takes place may also negatively influence an autistic child’s participation and experience.

Accordingly, OESA programs and program leaders should make efforts to create inclusive and accessible OESAs for autistic children. For example, program leaders should endeavour to offer clear yet flexible communication regarding the structure, expectations, and rules of the OESA to reduce uncertainty. Additionally, training OESA program leaders to understand and support the unique needs of autistic children could also aid in increasing OESA participation. Further, practitioners and OESA program leaders could encourage peer support and buddy systems to facilitate social interactions and friendships for autistic children participating in OESAs. Importantly, the roles that parents, peers, and program leaders have in creating safe and inclusive social environments for OESAs should be acknowledged. By advocating for the inclusion of autistic children in OESA programs that recognise the barriers to their participation, the social, developmental, and health benefits for autistic children may be increased.

## Conclusion

The present study highlights that OESAs designed to meet the unique requirements of autistic children are needed. Based on the current findings, it is plausible that autistic children could increase OESA participation if programs are designed to foster connectedness and reduce negative attitudes which parents perceived as barriers to participation. This is an important area for future interventions. More broadly, OESA programs and program leaders should be aware that, in addition to the child’s ability and behaviour, program and social factors may be significantly less positive for OESA participation amongst autistic children compared to their neurotypical peers. It is important for program leaders and other professionals to create an inclusive environment in OESAs that are welcoming and supportive of autistic children. Policies, training programs, and interventions that address social and program barriers for autistic children may increase the likelihood of sustained, positive OESA participation.

## Appendix

See Table 5 and 6

**Table 5:** Nationality, race, age and sex characteristics of the final sample of participants and the number of participants represented at each level of engagement

Nationality and Race Characteristics of the Final Sample of Participants		
Characteristic	Full Sample	
	*n*	%
Nationality		
Australian	179	86.89
British	9	4.37
New Zealand	4	1.94
American	2	0.96
Colombian	2	0.96
Singaporean	2	0.96
Dutch	1	0.49
French	1	0.49
Irish	1	0.49
Italian	1	0.49
Mexican	1	0.49
Pakistan	1	0.49
Vietnamese	1	0.49
Preferred not to respond	1	0.49
Race		
White	172	83.50
Asian	11	5.34
Aboriginal Australian	4	1.94
Latinx	4	1.94
African	1	0.49
Multiracial	1	0.49
Preferred not to respond	13	6.30

The Age and Sex of Autistic Children the Final Sample of Participants Represented										
	Age									
	4 years	5 years	6 years	7 years	8 years	9 years	10 years	11 years	12years	Total
Male	1	1	3	1	3	6	1	6	2	24
Female	2	1	0	1	2	1	2	2	0	11
Total	3	2	3	2	5	7	3	8	4	35

The Age and Sex of Neurotypical Children the Final Sample of Participants Represented										
	Age									
	4 years	5 years	6 years	7 years	8 years	9 years	10 years	11 years	12 years	Total
Male	15	7	8	10	8	11	11	9	3	82
Female	16	6	13	11	7	5	13	10	7	88
Other	0	0	0	0	0	0	0	0	1	1
Total	31	13	21	21	15	16	24	19	11	171

The Final Sample of Participants at Each Level of Engagement of the OESA-EQ			
	Level of Engagement		
	Level One	Level Two	Level Three
Autistic	34	30	24
Neurotypical	170	124	104
Total	204	154	128

*N *= 206, *Level One* = fully engaged, *Level Two* = partially engaged, *Level Three* = considered engagement

**Table 6:** Preliminary statistical information

Frequencies and Chi-Square results for parent-reported OESAs (Coded for Category) for both the autistic and neurotypical groups													
Level of Engagement	OESA Category										*χ*2(4)	*p*	*ϕ*_c_
	Religious		Academic		The Arts		Sports		Community/ Service				
	*Autistic*	Neuro-typical	*Autistic*	Neuro-typical	*Autistic*	Neuro-typical	*Autistic*	Neuro-typical	*Autistic*	Neuro-typical			
	(%)	(%)	(%)	(%)	(%)	(%)	(%)	(%)	(%)	(%)			
Level One	1 (2.9%)	1 (0.6%)	1 (2.9%)	1 (0.6%)	5 (14.7%)	46 (27.1%)	25 (73.5%)	113 (66.5%)	2 (5.9%)	9 (5.3%)	5.16	.272	.16
Level Two	0 (0.0%)	1 (0.8%)	0 (0.0%)	2 (1.6%)	5 (16.7%)	33 (26.6%)	22 (73.3%)	84 (67.7%)	3 (10.0%)	4 (3.2%)	4.24	.374	.17
Level Three	0 (0.0%)	0 (0.0%)	0 (0.0%)	3 (2.9%)	5 (20.8%)	28 (26.9%)	14 (58.3%)	65 (62.5%)	5 (20.8%)	8 (7.7%)	4.34	.227	.184

Level One = fully engaged, Level Two = partially engaged, Level Three = considered engagement
